# Nurturing wellbeing amidst the climate crisis: on the need for a focus on wellbeing in the field of climate psychology

**DOI:** 10.3389/fpsyg.2023.1205991

**Published:** 2023-07-27

**Authors:** Amy Isham, Gareth Morgan, Andrew Haddon Kemp

**Affiliations:** ^1^School of Psychology, Swansea University, Swansea, United Kingdom; ^2^Centre for the Understanding of Sustainable Prosperity, Guildford, United Kingdom; ^3^School of Psychology, University of Leicester, Leicester, United Kingdom

**Keywords:** wellbeing, sustainability, climate change, mental health, connectedness, post-growth, planetary wellbeing, collective wellbeing

## Abstract

Awareness of climate change can prompt overwhelming emotions that threaten wellbeing such as anger, despair, and anxiety. Neoliberal views of human beings and their mental health strip the individual from their social and material context, driving personal dissatisfaction, social isolation, and ecological destruction. In this piece, we contend that advancements in scholarly research on wellbeing offer valuable insights for addressing the challenges posed by the climate crises while respecting human wellbeing. Such frameworks, which include the Power Threat Meaning Framework (PTMF) and the GENIAL model, emphasize the interconnected nature of people, communities, and their environment. In turn, they help to lay the groundwork for the development of ‘post-growth’ societies focused on supporting outcomes such as human wellbeing, social justice, and environmental regeneration. There are a number of different actions that practitioners and even lay individuals can take to promote positive outcomes and effective responses in the face of the climate crisis. These actions, discussed in the concluding sections of the article, aim to foster wellbeing and impactful engagement with the challenges posed by climate change.

“… With a profound awareness of interconnection, he wept and wept until his grief became a deluge, frozen trauma unleashed. Resolution was beginning… “

—Wendy Greenspun (2023, p. 63) in ‘Climate, Psychology and Change’

## Introduction

The inspiration for this paper comes from a screening of the documentary “The Oil Machine”^1^ where an audience member expressed despair and apprehension and questioned if there was any hope for the future. Such reactions are common, with one recent large-scale survey of 10,000 young people across 10 nations reporting that 75% of their sample agreed that ‘the future is frightening’ and over half (56%) agreed that ‘humanity is doomed’ (Hickman et al., 2021). People employ a range of coping strategies, defenses, or threat responses to protect against emotional overwhelm and, although it is important to caution against labelling responses as ‘helpful’ or ‘unhelpful’, some defenses can pose additional threats to individuals, communities, and the planet (Andrews and Hoggett, 2019; Morgan et al., 2022).

Whilst the interconnectedness between individuals, their communities and the more-than-human-world is central to the thinking of many of the world’s cultures (Adams, 2021), dominant discourses around psychological wellbeing in westernized nations have traditionally emphasized the individual. This siloed approach props up neoliberal agendas, underpinned by the dominant hegemony of neoliberalism that is driving the climate and ecological emergencies (e.g., Adams et al., 2019; Stoddard et al., 2021; Weintrobe, 2021). Neoliberalism is a political and economic ideology, characterized by unbridled individualism, free market competition and limited public expenditure, that obscures the impact of politics, inequity, and environmental degradation on the wellbeing of individuals (e.g., Clegg and Lansdall-Welfare, 2021; Davies, 2022). However, ongoing developments within human-environment interactions and wellbeing science support recognition of the interconnectedness of different dimensions of wellbeing, encompassing the self, other people, and the planet (Batell and Adams, 2016; Andrews and Hoggett, 2019; Lomas et al., 2020; Mead et al., 2021; Kemp and Edwards, 2022; Woodbury, 2022).

In this piece, we contend that recent advancements in scholarly research on wellbeing can be meaningfully integrated into the field of climate psychology, an emerging field of psychology that examines the emotional and existential impacts of the unfolding climate crisis. We believe ideas within climate psychology compliment work from other fields including positive, community and environmental psychology, which have produced seminal work concerning individual, collective and planetary wellbeing (see Ryff, 1989; Diener, 2000; Capaldi et al., 2015; and Di Martino et al., 2017 for reviews), supporting understanding and effective responses to the unfolding climate crisis. Our paper begins by highlighting how traditional, western discourses of the nature of human beings and ‘mental illness’ drive personal dissatisfaction, social isolation, and ecological destruction. We then move our attention to consider how developments in psychology are providing alternative frameworks from which to consider the nature of distress and wellbeing that are rooted in an understanding of the interconnected nature of human beings and their environment. We end by outlining practical steps that can be taken, by practitioners or lay individuals, to encourage wellbeing and effective action in the face of the climate crisis.

## A disconnected view of human nature

Neoclassical economics, an approach that attempts to explain economic processes through supply and demand, is structured around a model of human beings known as ‘homo economicus’ (Melé and Cantón, 2014). Homo economicus characterizes people as self-interested, rational, and outcome-orientated (Gintis, 2000). That is, people only think and act according to their own interests, seeking to always obtain the greatest pleasure or benefit for themselves given the means at their disposal. They have been regarded as only caring about others’ welfare to the extent to which it affects their own (Urbina and Ruiz-Villaverde, 2019). The pursuit of self-interest was justified by political economists as a driver of economic activity. For instance, in his now famous passage from The Wealth of Nations (Smith, 1776), Adam Smith (1723–1790) noted that “It is not from the benevolence of the butcher, the brewer, or the baker, that we expect our dinner, but from their regard to their own interest. We address ourselves, not to their humanity but to their self-love, and never talk to them of our own necessities but of their advantages.” (Smith, 1776, p. 456).

More recently, the notion of human beings as homo economicus has been criticized as “inadequate and deficient in portraying the complexity of human behavior” (Urbina and Ruiz-Villaverde, 2019, p. 85). Nevertheless, the ‘norm of self-interest’ (Miller, 1999) persists to some extent, and the pursuit of individual wealth is still encouraged by advertising and marketing communications within consumer capitalist societies. A common consequence of such communications is strong and/or prevalent materialistic values, whereby individuals are socialized to consider their own financial and material wealth to be the key determinant of their happiness and status within society (Richins and Dawson, 1992; Weintrobe, 2021). Yet whilst materialism encourages the consumption behaviors that underpin consumer capitalist systems (Kasser, 2017), it is also known to be detrimental to individual, collective, and planetary wellbeing (Dittmar and Isham, 2022). People who hold strong materialistic values report poorer life satisfaction (Dittmar et al., 2014), lower mood, elevated anxiety (Bauer et al., 2012), and show less concern for the environment (Hurst et al., 2013). On a more macro level, the link between levels of consumption and wellbeing is also ambiguous. Neoliberal policies have resulted in heightened economic inequality, with the gap between the wealthiest and poorest in a nation being one of the strongest predictors of a whole range of health and social problems (Wilkinson and Pickett, 2010). Recent research has also documented that within countries experiencing consumption growth per capita, higher levels of consumption are not linked to greater happiness levels (Fanning and O'Neill, 2019). Accordingly, although growth in consumption and spending are often a key part of government policy, a preoccupation with accumulation of possessions can be detrimental to both people and the planet.

In line with an individualistic focus, positivist traditions in the discipline of psychology and psychiatry rarely attend to the economic, material, and political landscapes that are of central importance in shaping experience (Boyle, 2022). Western mental health care provision continues to be dominated by constructs of ‘mental disorder’ which not only lack empirical support (e.g., Timimi, 2013; Johnstone, 2022) but serve to prop up neoliberal agendas through reframing social problems as stemming from an individual’s biology or thinking style (Moncrieff, 2009; Harper, 2013; Davies, 2022; Elf et al., 2023).

## An alternative framework of distress

Based on a large body of evidence linking various forms of adversity and oppression to distress, the Power Threat Meaning Framework (PTMF; Johnstone and Boyle, 2018) was developed to offer an alternative conceptual lens for making sense of manifestations of distress. The Framework posits that various forms of power can pose threats to our core human needs (e.g., the need for physical safety, to have proximity to attachment figures, to have valued roles, to feel loved, etc.). In response to these threats, people react with various threat responses that are influenced by the meaning they attribute to the threats and the resources they have access to. Often these responses would be regarded as ‘symptoms’ of ‘mental illness’ from a medical model, but through the PTMF they are recognized as things people do, consciously or otherwise, to attempt to avoid or reduce the impact of threats. The Framework highlights that the threat responses available to a person will inevitably be constrained by the power resources they have access to (e.g., a person’s ability to take part in pro-environmental action will vary depending on financial resources and being able to take time out of work). The meanings a person ascribes to the threats can also influence their choice of threat response, and these meanings are shaped by socio-cultural operations of ideological power, including discourses concerning how people are ‘meant’ to think, feel, and behave in certain contexts (Boyle, 2022).

Recent work has explored how the PTMF can help us understand different reactions to climate breakdown, particularly in relation to the effects of economic, ecological, and ideological power on individuals and communities (Morgan et al., 2022). For instance, discourses that shift responsibility for change onto individuals instead of large corporations support the status quo and can result in threats to a person’s identity if a person develops meanings relating to guilt concerning their contributions towards emissions. Discourses that minimize the severity of the crisis or negatively frame activists can result in relational threats, such as alienation from peers and relatives who do not share concerns, or a sense of betrayal when they experience powerful others as failing to look after their interests. A concrete example of how the PTMF has been applied through a climate lens is the case study of the platinum mining town of Rustenburg, South Africa (Barnwell et al., 2020). Environmental-related psychological distress was contextualized through discussion of unequal power relations between the low-income Black community and other state and corporate actors, highlighting racial environmental injustices. Such power relations were characterized by ‘gaslighting’ those most vulnerable to the impacts of climate change by manipulating outcomes and delaying much-needed urgent action, while individuals in the community question their own sanity. This case study also highlighted the dangers of focusing on phrases like ‘climate anxiety’ without first contextualizing such experience within the context of asymmetrical power dynamics.

As threat responses are impacted by the power resources that an individual has available to them, the PTMF encourages individuals to locate and utilize their strengths (the power resources that they have available to them) to enable useful threat responses. These strengths can include involvement in social action or connecting with one’s values with an eye on positive change (Morgan et al., 2022). In this way, the PTMF provides a means of considering the nature of wellbeing as well as distress; highlighting some of the ways of encouraging resilience and effective action in the face of threats imposed by climate change.

## Toward a transdisciplinary theory of wellbeing

Wellbeing has been described as a “wicked problem” due to its complexity and lack of straightforward solutions (Bache et al., 2016). This has contributed to much debate and controversy in the field. The discipline of psychology has been criticized for abstracting the self from social and material contexts (e.g., Adams et al., 2019). According to this critique, psychology contributes to the reinforcement and dominance of neoliberal systems, which place the responsibility on individuals for generating positive outcomes in their lives, while attributing blame when negative circumstances arise (Boyle, 2022). Psychological techniques such as mindfulness have been commercialized for financial gain (e.g., ‘McMindfulness’, Purser, 2019) while the field of positive psychology has been likened to ‘glad games’ that Pollyanna played to cope with loss, abuse and prejudice, diverting attention away from underlying systemic issues, a critique referred to as ‘scientific Pollyanna-ism’ (Yakushko, 2019). Related criticisms point out that research on wellbeing has disregarded non-Western approaches (Bednarek, 2024) which emphasize the inter-relatedness between people, their communities and the more than human world. These critiques have highlighted a need for psychological scientists to move beyond disciplinary boundaries, and it is this background on which the landscape of positive psychology and the more heterogeneous research domain of wellbeing science has begun to change (Lomas et al., 2020; Kemp and Edwards, 2022).

**Figure 1:**
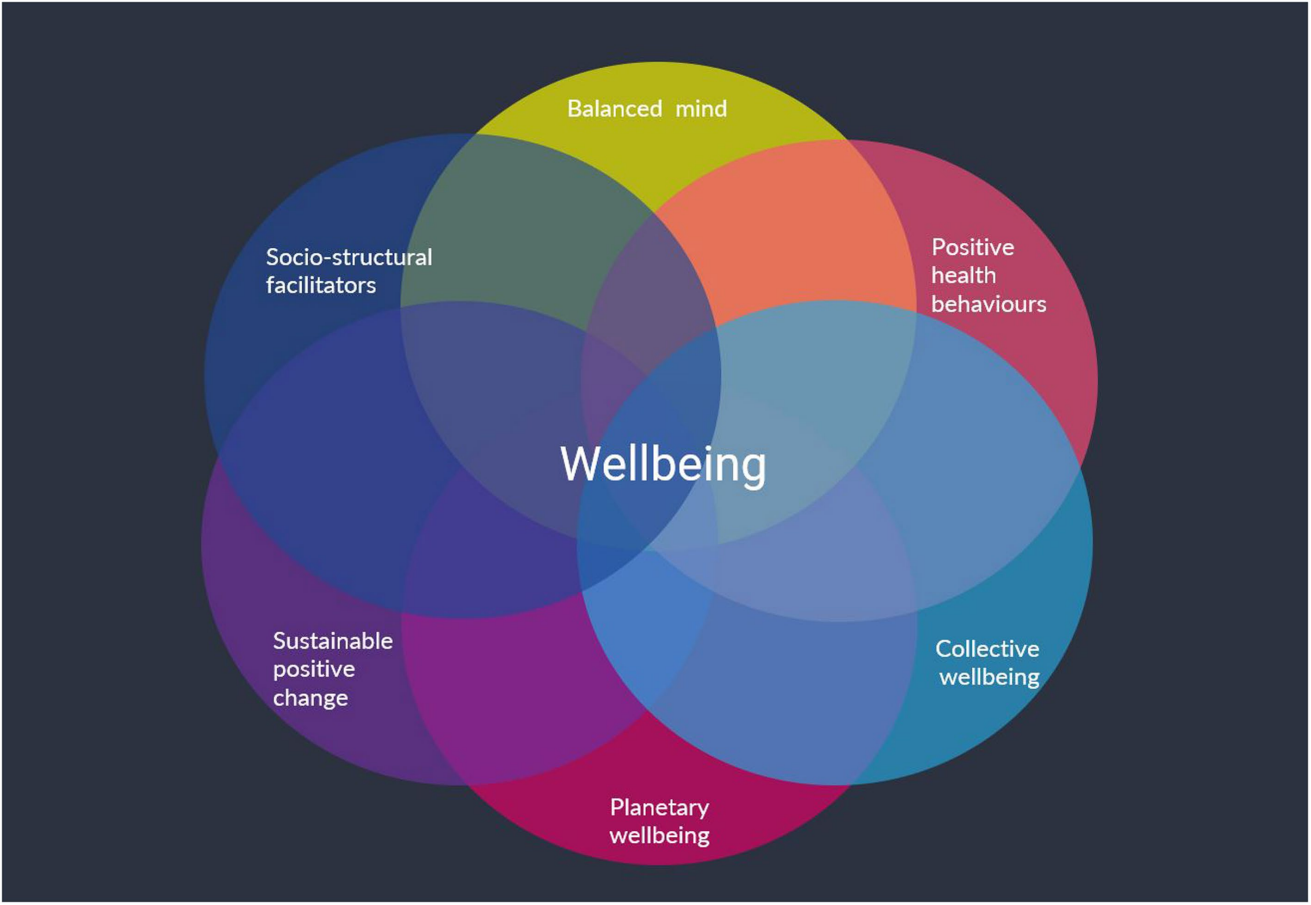
The core components of the GENIAL model, integrating often isolated insights that span across the domains of the individual, community, and the environment. The capacity for individuals to promote their own wellbeing is much greater than their capacity to promote collective wellbeing, which is greater than their capacity to promote planetary wellbeing. Yet, despite these constraints, there is tremendous potential for individuals to promote individual wellbeing while focused on collective and planetary wellbeing, by connecting to the self, others, and planet. For further details on this model and its development, interested readers are referred to the following articles: (Kemp et al., 2017; Fisher et al., 2020; Mead et al., 2021, 2023; Kemp and Fisher, 2022; Wilkie et al., 2022a).

Scholars are increasingly calling for the realization of *sustainable wellbeing* (Isham et al., 2022b; O'Mahony, 2022; Mead et al., 2023), *sustainable fulfilment* (Isham and Jackson, 2022), *sustainable happiness* (O’Brien, 2016), or *sustainable hedonism* (Lelkes, 2021), to name just a few. At the heart of all these terms is the possibility that high levels of human wellbeing can be achieved by reducing environmental impacts and increasing care for, and connection to, other people and the environment. Feeling a connection to nature, for example, has been shown to provide benefits for an individual’s happiness and meaning in life (Capaldi et al., 2014; Pritchard et al., 2020) plus prompt a greater prosocial orientation (Passmore and Holder, 2017), higher levels of environmental concern (Schultz et al., 2004) and engagement in pro-environmental behaviors (Martin et al., 2020), exemplifying potential co-benefits to individual, collective, and planetary wellbeing. It is against this background that the GENIAL model of wellbeing was developed (Kemp et al., 2017; Fisher et al., 2020; Mead et al., 2021, 2023; Kemp and Fisher, 2022; Wilkie et al., 2022a), motivated by the need for an interpretative framework that could bridge isolated lines of research, resulting from the multidisciplinary, multidimensional and multi-levelled body of work that has been conducted on this topic. The GENIAL model is focused on connections to the self, community, and nature, around which positive change has been facilitated at multiple levels of scale to promote wellbeing—broadly defined—with an eye on harnessing sociostructural opportunities (see Figure 1) such as individual and community strengths and resources, consistent with the focus of the PTMF.

A concrete example of how the GENIAL framework has been applied is a 5-week undergraduate student module focused on improving wellbeing in university students alongside collective and planetary wellbeing. In this module, students can personalize their approach to wellbeing promotion (Ciarrochi et al., 2022) by self-selecting various activities that span different components of wellbeing, ranging from the individual to the planet. They then write up the impact of these interventions on their own wellbeing in an N-of-1 case study (Kemp and Fisher, 2021). For instance, students might decide to focus on different types of meditation over the course of the module including a breath-focused meditation technique (e.g., Nestor, 2020) to promote self-connection (Klussman, 2021; Wilkie et al., 2021), a loving-kindness meditation (e.g., Hutcherson et al., 2008) to support social connection, and a nature-based meditation (e.g., Mortali, 2019) to support nature connection. In the final week, students reflect on positive behavioral change through a goal setting activity, a practice that has been shown to underpin individual wellbeing outcomes (e.g., Grégoire et al., 2012). A recent study highlighted the beneficial impacts that this module has had on student wellbeing (Kemp et al., 2022).

The GENIAL framework has also been applied to the healthcare sector, leading to a complete redesign and transformation of healthcare services in neurorehabilitation, in particular (Fisher et al., 2020; Gibbs et al., 2022a). For example, recently published research (Gibbs et al., 2022b; Wilkie et al., 2022b) has highlighted how nature-based interventions, such as surf therapy, can be used to facilitate aspects of wellbeing in people living with acquired brain injury (ABI) at multiple levels of scale. Surf therapy led to changes at the scale of (1) individual wellbeing—increased mindfulness and physical activity; (2) collective wellbeing—improved relationships, community participation, and contribution to organizations; and (3) planetary wellbeing—connection to nature with implications for pro-environmental behaviors (Wilkie et al., 2022b). Critically, actions to support individual and collective wellbeing were aligned with those required for planetary health and wellbeing, in line with prior research showing that nature connectedness supports wellbeing and helps facilitate pro-environmental behaviors (e.g., Martin et al., 2020). This work has special significance for the field of climate psychology, highlighting the opportunity for wellbeing alongside adversity, hardship and suffering, an approach central to the emerging field of existential positive psychology (Wong et al., 2021). By fostering a sense of connection with the self, others, and nature, in a systems-informed way (Kern et al., 2020; Gibbs et al., 2022a), individuals, groups, and organizations are laying the foundations for a ‘post-growth’ future that prioritizes wellbeing and sustainability over economic growth and material consumption.

## Crafting ‘post-growth’ futures

Post-growth envisions a society that is not dedicated to the pursuit of constant economic growth, but rather prioritizes outcomes such as human wellbeing, social justice, and environmental regeneration (Jackson, 2021; Paulson and Büchs, 2022). Several different theoretical proposals can be considered to represent post-growth futures in that they prioritize wellbeing over economic output, such as doughnut economics (Raworth, 2017), wellbeing economics (Costanza et al., 2018), and degrowth (Kallis, 2018). At the heart of post-growth thinking is the idea that human wellbeing does not have to be compromised by living in more sustainable and less resource-intensive ways (Jackson, 2005). Instead, human wellbeing is actually enhanced by increasing consideration for, and connection to, other people and the environment. The movement therefore draws upon ideas from environmental psychology concerning the interconnectedness of individual, collective and planetary wellbeing, but places them in a more macro context whereby they should form the basis of our economic systems and way of life.

Psychological research documents multiple ways in which feelings of connectedness can be enhanced (Isham et al., 2022a). These include experiences of awe that are commonly elicited by encounters with nature, arts, or extraordinary talent (Chirico and Yaden, 2018). During such experiences, individuals tend to feel small in comparison to the stimuli they are confronted with, leading them to direct their attention away from themselves and towards larger non-self entities (Shiota et al., 2007). Intense engagement with every-day activities that one intrinsically enjoys can lead to flow states (Csikszentmihalyi, 1992; Isham and Jackson, 2022), which are highly enjoyable and temporarily reduce self-consciousness whilst fostering feelings of connection to the activity. Isham et al. (2019) illustrated that flow experiences often occur in activities with low environmental costs, such as sports, arts, socializing, contemplative practices, and romantic relationships. They thus can offer a means of increasing wellbeing and feelings of connection in more environmentally friendly ways. There are parallels between such approaches and the ‘Work that Reconnects’ (Macy and Johnstone, 2022), which provides a therapeutic framework for cultivating connections with others and the more-than-human world. The ‘Work That Reconnects’ involves a series of practices that cultivate gratitude, and honor and process the pain about the state of the world, while facilitating a shift in perspective to recognize interconnections and exploring possibilities for positive change in personal lives, communities, and society for a more sustainable future. Mindfulness practices play a significant role within this framework, supporting a sense of embodied connection with the more-than-human world and future generations. Research has indicated that such practices enhance connectedness, improve wellbeing, promote pro-environmental attitudes, and encourage sustainable behaviors (Brown and Kasser, 2005; Sheth et al., 2011; Barbaro and Pickett, 2016; Wamsler et al., 2018; McKay and Walker, 2021; Schmid and Aiken, 2021; Tse et al., 2021; Zelenski and Desrochers, 2021). What is evident is that efforts to mitigate the effects of the climate crisis can be pursued in a way that values and supports human wellbeing alongside environmental concerns.

## Action to support wellbeing in a climate crisis

The socio-ecological challenges facing humans highlights an urgent need for transformative psychological approaches to manage overwhelming emotions that often arise in the face of climate change and encourage effective engagement with socio-ecological problems (e.g., Hickman et al., 2021). An emerging movement described as the inner development goals (IDGs) seeks to promote psychological skills and qualities to help facilitate progress towards the UN Sustainable Development Goals (SDGs). The IDGs include a focus on being (i.e., relationship to self), thinking (cognitive skills), relating (caring for others and the world), collaborating (social skills) and acting (driving change). A developing library of tools (see: https://idg.tools/) has been proposed to help foster the skills and qualities urgently needed for societal transformation. In Table 1 we expand on these to provide suggestions for how connection can be facilitated at individual, social, and planetary levels. We are aware that the IDGs could be interpreted as inconsistent with urgently needed eco-systemic approaches to managing the climate crisis. The IDGs initiative applies a Western scientific lens to the climate crisis, an approach that has traditionally excluded the wisdom of other cultures (e.g., the South African concept of ‘Ubuntu’, which has origins in the Zulu and Xhosa languages and is translated as “humanity towards others” or “I am because we are”; Topidi, 2022). We would counter this concern however by pointing out that the IDG framework is not necessarily inconsistent with an eco-systemic approach—that embraces different ways of knowing—as long as it is still based on evidence grounded in for example, critical realism, a philosophical framework that moves beyond scientific positivism by taking into account objective reality in addition to the subjective interpretations and constructions of that reality by individuals and social groups. In fact, Kemp’s work that has been conducted in the education and healthcare sectors (see Gibbs et al., 2022a and Fisher et al., 2020 for reviews) through the lens of the GENIAL model was motivated and inspired by an eco-systemic approach to building population wellbeing (see also Mead et al., 2023).

**Table 1:** Opportunities for promoting individual, collective and planetary wellbeing and laying the foundations for positive sustainable futures.

Domain	Key concepts	Strategies and potential benefits
Connecting to the self (“self-connection”)	Individual wellbeing may be defined as “self-connection” a concept that is rooted in the awareness of oneself, and the acceptance and alignment of behavior based on that awareness. Other relevant concepts include: self-determination, self-compassion, acceptance, personal values, learning mindset, self-care, character strengths, mind–body connection, breathwork, goal setting, psychological flow, and positive health behaviors.	Connecting to the self lays strong foundations for connecting to others and the environment. Activities include goal-setting exercises supporting capacity for self-determination, training in mindfulness to help increase awareness of one’s inner thoughts and feelings, and psychological flow, an experience associated with environmentally friendly activities. Mind–body practices such as breathwork and the martial arts help individuals build ‘fierce compassion’, and to better regulate their emotions and navigate conflict. Positive health behaviors including a focus on diet, exercise and sleep are also essential for health and wellbeing. Climate-informed psychotherapy helps individuals and communities to manage distress arising from climate breakdown.
Connecting to others (social connection)	Collective wellbeing may be defined as the interdependence of ‘feeling good’ and functioning well. Other key concepts include social-relational emotions, kindness, compassion and care for others, interpersonal trust and psychological safety, relationship building, positive relationships, social capital and social identity. Co-creation, partnership working and strategic partnerships facilitate a systems-informed approach to collective wellbeing.	Connecting to others supports individual wellbeing but also facilitates empowerment, agency and social action. Social connection may be facilitated through—for example—compassion training, volunteering activities, group-based activities (e.g., arts, crafts, sports), ‘Prosocial’ practices for more equitable collaboration, and participation in different forms of activism, promoting a sense of meaning and purpose, and self-transcendence. Communal psychotherapeutic practices including ‘climate cafes’, ‘warm data labs’ and ‘Radical Joy for Hard Times’ provide a space for group working and processing of difficult emotions. The inner development goals also seek to promote effective communication and mobilization skills.
Connecting to the environment (nature connection)	Planetary wellbeing is arguably the highest attainable standard of wellbeing that supports individual and collective wellbeing in a systems-informed way. Other key concepts include the expansion of self to non-human others, (re)connection to nature, respect and care for the environment, meaning and purpose, active hope, sustainable and ecological wellbeing, and self-transcendent experience.	Connecting to nature lays foundations for transformative change, by not only promoting wellbeing, but also fostering pro-environmental behaviors. Activities include for example, ‘imagination activism’, an IDG technique that involves imagination exercises and meditation, support individuals to explore alternative worlds and possibilities, inspiring hope. Other activities include the ‘Three Good Things’ in nature exercise, forest bathing (also known as ‘shinrin-yoku’), and nature-based mindfulness such as the ‘Breath, Relax, Feel, Watch, Allow’ (BRFWA) routine, all of which facilitate sustainable wellbeing. Psychedelic experience may also promote ecological wellbeing and facilitate self-transcendent experience.

These strategies serve as a starting point for considering how to enhance wellbeing while also facing down the climate crisis. Our table draws on ideas from a range of sources including the GENIAL theoretical framework (Mead et al., 2021, 2023; Kemp et al., 2022; Kemp and Fisher, 2022; Wilkie et al., 2023), which has sought to integrate the different aspects of individual, collective and planetary wellbeing, drawing on the work of Allison et al. (2021), Antó et al. (2021) and Klussman (2021), respectively. The development of the GENIAL model was motivated by the need to apply an interpretative framework to an often-contradictory body of multidisciplinary, multidimensional and multi-levelled research on wellbeing. This table also draws on the inner development goals and tools (Stålne and Greca, 2022; see also: https://www.innerdevelopmentgoals.org/ and https://idg.tools/), an emerging framework and library of tools for accelerating progress on the UN Sustainable Development Goals. Prosocial practices are inspired by Atkins et al. (2019); communal psychotherapeutic practices are drawn from Bednarek (2024); the ‘Three Good Things’ in nature exercise and BRFWA routine are described in Keenan et al. (2021) and Mortali (2019), respectively. Although specific strategies have been outlined for each domain, it is also important to note that many of these strategies are interconnected and can support growth across multiple areas.

## Conclusion

In conclusion, the mainstream discourse around psychological wellbeing has traditionally focused on individual wellbeing, often overlooking the interdependence of individual, collective, and planetary wellbeing. The GENIAL model and the PTMF both draw on themes from positive, community and environmental psychology and support a broader perspective of wellbeing that acknowledges the interconnectedness of people and planet, as well as opportunities for positive societal change. These frameworks, and other approaches that have come before (e.g., Macy and Johnstone, 2022), not only support orientation towards making sense of wellbeing within social, political, and ecological contexts, but encourage focus on individual and community strengths. What these developments suggest is that, alongside acknowledging and managing overwhelming responses to the climate crisis, we can also work on building individual and collective resources that can support the development of ‘post growth’ societies and thus wellbeing at a personal, social, and planetary level. In other words, there is space to experience renewed purpose through engaging with the crises that we collectively must face (Macy and Johnstone, 2022; Samuel et al., 2022). Indeed, proponents of post-growth thinking recognize a need to avoid being dragged into purely hedonistic pursuits, and place importance on being able to live with and utilize the power of uncomfortable states (Isham et al., 2021; Jackson, 2021). We suggest that psychologists have a key role to play in facilitating individual capacities for wellbeing alongside suffering by promoting inner development through eco-systemic evidence-based interventions, supported by strategic partnerships and collaborative work on which system-wide change can be achieved (e.g., Atkins et al., 2019; Gibbs et al., 2022a; Bednarek, 2024).

We propose that the current state of climate-focused psychology presents a similar opportunity to the one Martin Seligman encountered in 1999 when he initiated positive psychology in his APA presidential address (Seligman, 1999). In that speech, Seligman urged the field of psychology to shift its focus towards the promotion of wellbeing and nurturing of human strengths, rather than exclusively attending to pathology. By drawing parallels to Seligman’s vision, we aim to help move climate-focused psychology beyond a prevailing emphasis on alleviating climate-related distress and grief. By highlighting the potential for wellbeing alongside suffering (Wong, 2019; Wong et al., 2021) we hope to prompt a shift in focus to undertaking the transformative changes required (Atkinson and Jaquet, 2022) for a post-growth future.

## Data availability statement

The original contributions presented in the study are included in the article/supplementary material, further inquiries can be directed to the corresponding authors.

## Author contributions

All authors listed have made a substantial, direct, and intellectual contribution to the work and approved it for publication.

## Funding

We acknowledge the financial support from the Centre for the Understanding of Sustainable Prosperity (CUSP), grant number: ES/T014881/01, which funds some of AI research time.

## Conflict of interest

The authors declare that the research was conducted in the absence of any commercial or financial relationships that could be construed as a potential conflict of interest.

## Publisher’s note

All claims expressed in this article are solely those of the authors and do not necessarily represent those of their affiliated organizations, or those of the publisher, the editors and the reviewers. Any product that may be evaluated in this article, or claim that may be made by its manufacturer, is not guaranteed or endorsed by the publisher.

## Footnotes

^1^ https://www.theoilmachine.org/
